# Association between dietary insulin index and postmenopausal osteoporosis in Iranian women: a case-control study

**DOI:** 10.1186/s12905-024-03248-z

**Published:** 2024-07-15

**Authors:** Shakiba Solgi, Farid Zayeri, Behnood Abbasi

**Affiliations:** 1 Department of Nutrition, Electronic Health and Statistics Surveillance Research center, Science and Research Branch, Islamic Azad University, Tehran, Iran; 2 Proteomics Research Center, Department of Biostatistics, School of Allied Medical Sciences, Shahid Beheshti University of Medical Sciences, Tehran, Iran; 3 Department of Nutrition, Electronic Health and Statistics Surveillance Research center, Science and Research Branch, Islamic Azad University, Hesarak Blvd, Daneshgah Square, Sattari Expressway, Tehran, Iran

**Keywords:** Osteoporosis, Insulin resistance, Hyperinsulinism, Insulin, Bone resorption, Postmenopausal women

## Abstract

**Background:**

The relationship between the dietary insulin index (DII) and the disease’s risk is unknown, despite the fact that hyperinsulinemia is presumed to contribute to osteoporosis. The insulin response of various diets determines the DII. This study aimed to investigate the connection between postmenopausal Iranian women’s adherence to a diet with a higher insulinemic potential and osteoporosis.

**Methods:**

A total of 380 postmenopausal women were included in the current case-control study. A 168-item food frequency questionnaire (FFQ) with established validity and reliability was used to evaluate individuals’ daily calorie intake. The standard formula was employed to determine the dietary insulin load of each product. Subsequently, the calculation of DII was performed by dividing the dietary insulin load by the total energy consumed for each individual. In order to investigate the relationship between osteoporosis and DII, logistic regression was implemented.

**Results:**

The results of the current study demonstrated a substantial inverse relationship between osteoporosis and the DII, even after accounting for confounding variables (OR = 0.927; 95% CI = 0.888–0.967). The mean scores of DII (*P* < 0.001) was significantly higher in control group (36.82 ± 8.98) compared to the case group (33.53 ± 6.28).

**Conclusions:**

Our findings suggest that keeping a diet high in insulin index and low in foods that are insulinogenic may improve bone mass density. Consequently, it may be essential for postmenopausal women to consume nutrients that stimulate insulin production in order to prevent osteoporosis.

## Introduction

The most common bone disease is osteoporosis [1], which causes a reduction in bone mineral content and an increase in bone fractures [2]. One in three women and one in five men over the age of 50 are at risk of fractures associated to osteoporosis, according to a research from the International Osteoporosis Foundation [3]. National Osteoporosis Prevention, Diagnosis, and Treatment Program in Iran has revealed that 50% of men and 70% of women over the age of 50 are affected by osteoporosis and osteopenia [4]. This disease is four times more prevalent in women during menopause than in men [5]. After menopause, Iranian women exhibit osteopenia and osteoporosis of lumbar spine at rates of 50% and 26.7%, respectively [6].

Previous research demonstrated that insulin could have anabolic effects on bone [7]. Additionally, serum glucose and insulin concentrations were positively correlated with bone mass and a reduced fracture rate [8]. According to the findings of some research, insulin hypersecretion and postprandial hyperinsulinemia may eventually develop in insulin resistance (IR) [9], which may then benefit bone mineral density (BMD) [8, 10, 11]. IR and BMD do, however, have a complex relationship [12]. Previous research [13] showed that there was a direct correlation between a higher IR and a higher dietary insulin index. The results of this study indicated that the Iranian population’s IR can be elevated by insulinogenic foods, such as rice and bread, which comprise the majority of their diet [13].

The postprandial insulin secretion of a variety of meals was estimated using the food insulin index (FII) [14]. In fact, the dietary insulin index (DII) may be computed using this metric [15]. Compared to the glycemic index (GI), which only communicates specific information about the carbohydrate content of meals and glycemic response [16], the DII is more appropriate for explaining the emergence of chronic diseases since it is accurately calculated based on insulin response [13]. Furthermore, the GI failed to effectively reflect the insulin response of a large number of meal components [16], but DII predicted insulin responses to mixed meals more accurately. There are contradicting studies regarding the impact of IR on BMD. Although certain studies have demonstrated a positive correlation between IR and BMD [8, 10, 11], others have failed a significant association between them [17]. The conflicting results compelled us to reassess their correlation. The objective of this study was to examine the correlation between Dietary Inflammatory Index (DII) and the susceptibility to osteoporosis in postmenopausal women from Iran. Because DII is properly determined based on insulin response rather than other mediators, it is more appropriate indicator than GI to predict the probabilities of chronic illness [18]. We postulated that insulin may stimulate bone growth and an increase in bone density. In the case that a significant negative association between DII and the incidence of osteoporosis were discovered, we would conclude that eating meals with high insulin index would be essential for postmenopausal women to avoid osteoporosis.

## Methods

### Study population

This case-control study recruited 380 postmenopausal women (190 cases and 190 controls) between the ages of 45 and 85 who were referred to Shariati hospital and several private clinics in Tehran, Iran. The rheumatology specialist’s diagnosis was established on the basis of the identification of osteoporosis using dual-energy X-ray absorptiometry [19]. The control group was selected from the visitors and patients’ companions who traveled to these institutions from various locations in Tehran and did not have any familial ties to the patients. Cases and controls were age- matched, and the participants in both groups were women who were postmenopausal, defined as having not had a menstrual period for at least 12 months. All participants refrained from using any drugs or dietary supplements that affect bone metabolism, including glucocorticoids, calcitonin, thyroxin, anticoagulants, antacids, calcium (500 mg/day), vitamin D (15 IU/day), glucosamine, or bisphosphonates. Additionally, they have not been diagnosed with any conditions that might affect their BMD status, such as renal, gastrointestinal, endocrine, or, rheumatic disorders. Moreover, they followed no specific diet throughout the previous year. Those who reported an inappropriate energy intake (< 800 kcal/day or > 4200 kcal/day) and those who did not answer more than 20% of the food frequency questionnaire (FFQ) items were excluded [20]. The ethics committee of the Islamic Azad University’s Science and Research Branch in Tehran, Iran, gave its approval for this work (IR.IAU.SRB.REC.1396.119).

### Study variables

Body weight was measured with a digital scale (Tefal) with an accuracy of 0.1 kg while wearing light clothes, and height was measured using a tape meter with an accuracy of 0.1 cm. The anthropometric measurements were body mass index (BMI), which was determined using the following formula: BMI, weight (kg), and height (cm). BMI is equal to body weight (kg)/ [height (m)]^2^. The survey collected information on many factors like age (in years), level of education (under graduate/ graduate/ post graduate), breastfeeding status (yes/no), alcohol consumption (yes/no), weight (in kilogram), duration of lactation (in weeks), and use of oral contraceptives (yes/no). Additionally, a valid questionnaire [21] was implemented to evaluate the extent of physical activity. Its validity was verified by the “CSA Accelerometer Ambulatory Monitor” system (Model 7164) and the Daily Activity Questionnaire, which were administered to 2500 Danish men and women between the ages of 20 and 60 [21]. The validity and dependability of the calculation were verified in Iranian women [22], and the amount of physical activity was determined using metabolic equivalent hours per week [21]. According to their levels of physical activity, participants in the present research were divided into three groups: very low (less than 600 MET-minutes per week), low (between 600 and 3000 MET-minutes per week), moderate and high (greater than 3000 MET- minutes per week) [23]. Participants’ dietary consumption was recorded using a 168-item FFQ, which is a valid and trustworthy tool [24, 25]. Next, the frequency of intake for each food item in the FFQ was translated to grams per day using household measurements [26]. Subsequently, the Nutritionist IV program was used to compute the quantity of macronutrients and micronutrients consumed.

### Assessment of dietary insulin index

By dividing the area under the insulin response curve for 1000 kcal of the test meal after two hours by the area under the curve for 1000 kcal of glucose, the reference food, the food insulin index (FII) was determined. The insulin index of 68 food items was obtained from previous research [14, 27, 28]. Tea, coffee, and sodium were allocated an insulin index of 0 as a result of their negligible caloric and macronutrient levels. Additionally, the food insulin index of comparable items was implemented in the event that specific items were not included in the designated food list. For example, dates and raisins, which are classified as dried fruits, have comparable energy, carbohydrate, fat, protein, and fiber profiles. As a result, for dates, we used the insulin index of raisins as a guide. First, we used the following formula to determine the insulin load of each meal: Insulin load of a certain food = Insulin index of the food × calorie content per 1 g of that food × quantity of that food eaten [29]. The DIL for each person was then determined by adding up the insulin load of every dietary item. We further calculated each person’s DII by dividing DIL by total energy used.

### Sample size calculation

A total of 176 samples were generated using GPower 3.1.9.2 (Kiel University, Germany) to analyze the sample size with α = 0.05, 95% power (β = 0.05), and an effect size of 0.1. In order to account for a 10% dropout rate, each group consisted of 190 people.

### Statistical analysis

All statistical analyses were conducted using SPSS software, version 26, from the IBM Corporation, Amonk, NY, USA. The independent sample t-test was used to evaluate the mean of normally distributed qualitative variables across two groups, and one-way ANOVA was utilized to compare the mean of normally distributed quantitative variables across more than two groups. Furthermore, we employed the Mann-Whitney test to investigate the differences in non-normal variables between the two groups. The Chi-squared test was implemented to evaluate the correlation between categorical variables in the control and case groups. The frequency distribution indices were employed to elucidate the qualitative data, while the mean and standard deviation (SD) were computed to represent quantitative variables. The odds ratio (OR) and 95% confidence intervals (CI) were calculated using binary logistic regression after adjusting for physical activity, BMI, and alcohol use to assess the relationship between the DII and osteoporosis. All analyses were considered significant when *P* < 0.05.

## Results

The demographic differences between the case and control groups are shown in Table 1. There was no discernible difference in the mean age of the two groups (55.99 ± 6.73 vs. 55.58 ± 6.07 years, *P* = 0.533). The mean DII in the control group was much greater (*P* < 0.001). The mean DII score for the case group was 33.53 ± 6.28, whereas the control group’s was 36.82 ± 8.98. Furthermore, the control group’s mean values for breastfeeding duration (*P* = 0.042) and physical activity (*P* < 0.001) were much greater. The control group’s weight was lower (*P* = 0.013), but their BMI was not different (*P* = 0.139). The alcohol consumption of cases was greater than that of controls (*P* < 0.001). The results shown in Table 2 show that there was no difference in the baseline characteristics across DII tertiles.

**Table 1:** Demographic characteristics of the participants in the case and control groups

Variables	Case (*n* = 190)	Control (*n* = 190)		*P*-value ^a, b^
Age (years)	55.99 ± 6.73*	55.58 ± 6.07		0.533
Physical activity (METs.h/day)	1488.21 ± 808.87	2265.94 ± 2142.61		< 0.001
Weight (kg)	74.03 ± 11.56	71.28 ± 9.89		0.013
BMI (kg/m^2^)	28.87 ± 4.09	28.12 ± 5.59		0.139
Duration of lactation (week)	29.40 ± 26.29	35.57 ± 32.41		0.042
Education				0.228
Undergraduate	154 (81.1)**	140(73.7)		
Graduate	34 (17.9)	47 (24.7)		
Postgraduate	2 (1.1)	3 (1.6)		
Breastfeeding				0.102
Yes	174 (91.6)	164 (86.3)		
No	16 (8.4)	26 (13.7)		
OCP				0.467
Yes	68 (35.8)	61 (32.1)		
No	122 (64.2)	128 (67.4)		
Alcohol use				< 0.001
Yes	26 (13.7)	5 (2.6)		
No	164 (86.3)	185 (97.4)		

*Mean ± SD, ** No (%)

Abbreviations: MET, metabolic equivalent; kg, kilogram; BMI, body mass index; kg/m^2^, kilogram per square meter; OCP, oral contraceptive pill.

^a^ Independent sample t-test was used for continuous variables and chi-square was used for categorical variables

^b^* P*-value less than 0.05 was considered significant.

**Table 2:** General characteristics of study participants in different tertiles (T) of the DII

	Total Mean ± SD or % (*n* = 380)	Tertiles of DII			
		T1 (*n* = 125)	T2 (*n* = 126)	T3 (*n* = 129)	*P* value^a^
Age (years)	55.79 ± 6.40	55.70 ± 5.83	55.79 ± 6.28	55.88 ± 7.06	0.977
Physical activity (METs.h/day)	1877.08 ± 1663.50	1753.92 ± 1170.24	2036.64 ± 2453.71	1840.56 ± 972.67	0.386
Weight (kg)	72.65 ± 10.83	74.32 ± 11.53	72.00 ± 10.74	71.67 ± 10.09	0.107
BMI (kg/m^2^)	28.49 ± 4.91	28.87 ± 4.37	28.02 ± 4.29	28.59 ± 5.87	0.381
Duration of lactation (week)	32.48 ± 29.63	35.26 ± 30.37	31.71 ± 26.93	30.55 ± 31.39	0.421
Education (%)					0.138
Undergraduate	77.4	82.4	75.4	74.4	
Graduate	21.3	15.2	23.0	25.6	
Postgraduate	1.3	2.4	1.6	0.0	
Breastfeeding (%)					0.377
Yes	88.9	88.0	92.1	86.8	
No	11.1	12.0	7.9	13.2	
OCP (%)					0.228
Yes	33.9	27.2	37.3	37.2	
No	65.8	72.0	62.7	62.8	
Alcohol use (%)					0.379
Yes	8.2	6.4	7.1	10.9	
No	91.8	93.6	92.9	89.1	
Physical activity (%)					0.086
Very low	16.1	20.8	16.7	10.9	
Low	74.2	72.8	69.8	79.8	
Moderate and high	9.7	6.4	13.7	9.3	
BMI (%)					0.094
Underweight	0.3	0.0	0.8	0.0	
Normal	17.4	19.4	18.5	14.3	
Overweight	48.7	38.7	51.6	55.6	
Obese	33.7	41.9	29.0	30.2	

Abbreviations: MET, metabolic equivalent; kg, kilogram; BMI, body mass index; kg/m^2^, kilogram per square meter; OCP, oral contraceptive pill

^a^ One-way ANOVA was used for continuous variables

The average consumption of nutrients in the two groups is shown Table 3. The control group exhibits considerably higher mean intakes of antioxidant vitamins and minerals, including vitamin A (*P* = 0.026), selenium (*P* = 0.002), alpha-carotene, beta-cryptoxanthin, vitamin C, zinc, and manganese (*P* < 0.001).

**Table 3:** Daily intake of nutrients in the case and control group

Nutrients	Case (190)	Control (190)	*P*-value ^a^
Energy intake (Kcal/day)	2675.32 ± 879.11*	2622.42 ± 875.01	0.557
Protein (gr/day)	82.68 ± 25.49	92.66 ± 31.57	0.001
Carbohydrate (gr/day)	345.82 ± 107.53	371.03 ± 122.98	0.034
Total fat (gr/day)	106.72 ± 45.47	80.79 ± 31.44	< 0.001
Cholesterol (mg/day)	259.76 ± 156.03	305.94 ± 441.24	0.175
Saturate fatty acid (gr/day)	32.15 ± 15.07	27.92 ± 16.76	0.010
MUFA (gr/day)	37.03 ± 16.42	28.77 ± 17.09	< 0.001
PUFA (gr/day)	23.28 ± 11.12	18.16 ± 10.38	< 0.001
Vitamin A (RAE/day)	676.21 ± 423.49	829.99 ± 845.72	0.026
Alpha-carotene (mg/day)	442.99 ± 728.45	1042.09 ± 1263.02	< 0.001
Beta-cryptoxanthin (mg/day)	128.92 ± 129.93	366.08 ± 364.81	< 0.001
Vitamin C (mg/day)	93.75 ± 68.48	183.62 ± 180.23	< 0.001
Vitamin E (mg/day)	15.88 ± 6.08	13.23 ± 7.17	< 0.001
Vitamin D (µg/day)	2.49 ± 1.99	2.72 ± 2.87	0.359
Calcium (mg/day)	1014.21 ± 403.40	1323.22 ± 722.21	< 0.001
Phosphorus (mg/day)	1506.52 ± 488.71	1964.58 ± 1049.22	< 0.001
Magnesium (mg/day)	396.97 ± 133.61	564.22 ± 334.38	< 0.001
Zinc (mg/day)	11.89 ± 3.85	15.47 ± 8.96	< 0.001
Manganese (mg/day)	6.61 ± 2.65	9.12 ± 5.59	< 0.001
Selenium (mg/day)	134.42 ± 55.69	161.95 ± 105.20	0.002

*Mean ± SD

Abbreviations: Kcal, kilo calorie; gr, gram; mg, milligram; µg, microgram; MUFA, monounsaturated fatty acid; PUFA, polyunsaturated fatty acid; RAE, retinol activity equivalent

^a^ Independent sample t-test was used for continuous variable

Table 4 describes the nutritional consumption among the various DII tertiles. Table 4 shows that there are no appreciable variations in the majority of nutrient intakes between the top and lowest tertiles of the DII.

**Table 4:** Dietary intake of nutrients in different tertiles (T) of the DII

Nutrients	Total Mean ± SD (*n* = 440)	Tertiles of DII			*P*-value ^a^
		T1 (*n* = 125)	T2 (*n* = 126)	T3 (*n* = 129)	
Energy intake (Kcal/day)	2648.87 ± 876.30	2887.86 ± 854.90	2579.90 ± 858.44	2484.66 ± 870.87	0.001
Protein (gr/day)	87.67 ± 29.09	92.36 ± 28.82	86.26 ± 28.21	84.51 ± 29.83	0.079
Carbohydrate (gr/day)	358.43 ± 116.05	363.99 ± 113.01	341.13 ± 105.62	369.93 ± 127.08	0.113
Total fat (gr/day)	93.75 ± 41.14	116.25 ± 42.21	91.03 ± 36.54	74.61 ± 33.31	< 0.001
Cholesterol (mg/day)	282.85 ± 331.31	291.50 ± 162.90	277.42 ± 161.74	279.78 ± 523.10	0.937
Saturate fatty acid (gr/day)	30.04 ± 16.06	36.57 ± 13.99	29.42 ± 13.84	24.32 ± 17.65	< 0.001
MUFA (gr/day)	32.90 ± 17.24	41.02 ± 15.86	31.19 ± 13.32	26.70 ± 18.89	< 0.001
PUFA (gr/day)	20.72 ± 11.04	25.61 ± 11.94	19.47 ± 8.66	17.21 ± 10.60	< 0.001
Vitamin A (RAE/day)	753.09 ± 672.34	839.01 ± 556.34	740.61 ± 461.39	682.04 ± 904.59	0.172
Alpha-carotene (mg/day)	742.54 ± 1072.42	608.27 ± 922.41	837.74 ± 1066.84	779.67 ± 1200.71	0.212
Beta-cryptoxanthin (mg/day)	247.50 ± 298.14	180.74 ± 209.40	265.72 ± 279.56	294.40 ± 370.80	0.007
Vitamin C (mg/day)	138.69 ± 143.40	117.58 ± 98.88	140.20 ± 108.03	157.66 ± 198.03	0.083
Vitamin E (mg/day)	14.56 ± 6.77	16.77 ± 6.37	13.93 ± 5.45	13.02 ± 7.73	< 0.001
Vitamin D (µg/day)	2.60 ± 2.47	3.18 ± 2.40	2.68 ± 2.20	1.97 ± 2.64	< 0.001
Calcium (mg/day)	1168.71 ± 604.31	1241.40 ± 561.25	1174.10 ± 513.85	1093.02 ± 712.27	0.146
Phosphorus (mg/day)	1735.55 ± 484.92	1787.99 ± 630.98	1663.02 ± 631.42	1755.58 ± 1162.14	0.481
Magnesium (mg/day)	480.60 ± 267.71	478.68 ± 188.73	449.27 ± 189.86	513.06 ± 374.79	0.163
Zinc (mg/day)	13.68 ± 7.12	13.96 ± 5.40	13.03 ± 5.20	14.04 ± 9.73	0.451
Manganese (mg/day)	7.87 ± 4.55	7.72 ± 3.33	6.82 ± 3.52	9.03 ± 5.99	< 0.001
Selenium (mg/day)	148.19 ± 85.18	153.66 ± 60.99	129.20 ± 61.60	161.43 ± 116.67	0.007

Abbreviations: Kcal, kilo calorie; gr, gram; mg, milligram; µg, microgram; MUFA, monounsaturated fatty acid; PUFA, polyunsaturated fatty acid; RAE, retinol activity equivalent

^a^ One-way ANOVA was used for continuous variables

After accounting for physical activity, weight, the duration of lactation, and alcohol consumption, Table 5 demonstrates a significant negative correlation between the DII and osteoporosis. The risk of osteoporosis decreased at a rate of 7% per unit increase in the DII (OR = 0.927; 95% CI = 0.888–0.967), as indicated in Table 5.

**Table 5:** Logistic regression findings for evaluating the relationship between the DII score and osteoporosis

Variable	Case (190)	Control (190)	Adjusted OR ^a^ (95% CI)	*P*-value ^b^
DII	33.53 ± 6.28*	36.82 ± 8.98	0.927 (0.888–0.967)	< 0.001

* Mean ± SD

Abbreviations: DII, dietary insulin index; OR, odds ratio; CI, confidence interval

^a^ Based on logistic regression adjusted for physical activity, weight, duration of lactation, and alcohol consumption

^*b*^ *Independent sample t-test was used for continuous variable*

Table 6 shows the relationship between DII tertiles and osteoporosis in both crude and adjusted models. According to the crude model, there was no significant correlation between people in the first and third tertiles of DII scores (crude OR = 1.35, 95% CI: 0.82–2.21, *P* = 0.240), but the odds of osteoporosis for women in the second tertile of DII scores were approximately 2.22 times higher than those in the third tertile (crude OR = 2.22, 95% CI: 1.35–3.67, *P* = 0.002). Furthermore, although there was no significant correlation between individuals in the first and third tertiles of DII scores (adjusted OR = 1.47, 95% CI: 0.87–2.49, *P* = 0.154), the adjusted model showed that the probabilities of osteoporosis for those in the second tertile were almost 2.32 times higher than those in the third (adjusted OR = 2.32, 95% CI: 1.36–3.97, *P* = 0.002).

**Table 6:** Logistic regression results for assessing the association between the DII tertiles and risk of osteoporosis

variable	category	Crude OR ^a^ (95% CI)	*P*-value ^b^	Adjusted OR ^c^ (95% CI)	*P*-value ^b^
DII tertiles	1	1.35 (0.82–2.21)	0.240	1.47 (0.87–2.49)	0.154
	2	2.22 (1.35–3.67)	0.002	2.32 (1.36–3.97)	0.002
	3	Ref.category			

Abbreviations: DAI = dietary antioxidant index; OR = odds ratio; CI = confidence interval

^**a**^ Based on logistic regression

^**b**^*P*-value less than 0.05 was considered significant

^c^ Based on logistic regression adjusted for physical activity, body mass index and alcohol consumption

## Discussion

According to the results of the present research, maintaining a diet with a high insulin index may improve bone mass density. In this work, we examined the relationship between the risk of osteoporosis and the dietary insulin score. Due to its reliance on insulin response rather than other factors, DII is a more accurate predictor of the probability of acquiring chronic disease compared to GI [18]. Previous studies indicated a somewhat advantageous correlation between DII and the probability of IR [13]. Our results demonstrate that the controls follow a diet with a higher tendency for insulinemia, as seen by the significantly higher mean DII in this group. This postprandial hyperinsulinemia may occur in IR gradually [9]. Furthermore, past studies have shown a positive association between IR and BMD [8, 10, 11]. Bazic et al. [10] examined the correlation between IR and BMD in postmenopausal women from Serbia. A positive connection was found between HOMA-IR and BMD-spine as well as bone mineral content. Furthermore, IR positively affects volumetric bone mineral density, as shown by a cross-sectional study [11] on postmenopausal Caucasian women. Additionally, the results of a cohort research [8], including close to 6000 senior men and women demonstrated a significant correlation between increased insulin and blood glucose levels and increased bone density and a decreased fracture risk. The relationship between IR and BMD is rather intricate. As a result, the processes that determine their precise relationship are not well known. Insulin may have anabolic effects on bone based on the research that is currently available [7]. The insulin receptors are expressed on the surface of osteoblasts and osteoclasts alike. Insulin is an anabolic hormone that has been shown in experimental investigations to support osteoblast growth [30]. Moreover, insulin-like growth factor 1 (IGF-1) receptors that are found on osteoblasts may be the source of this. Consequently, an increase in BMD may result from bone formation [31]. The inverse association between sex hormone-binding globulin (SHBG) and insulin is another likely example of a greater BMD in those with higher insulin levels [32]. Then, a drop in SHBG levels causes a rise in free estrogen concentration, which raises BMD [33].

In addition, prior research has shown the anabolic function of insulin in bone and established that physiological insulin concentrations may stimulate osteoblast growth, glucose uptake, and inhibit osteoclast activity [34]. Based on our research, the control group’s mean intake of protein and carbohydrates is significantly higher than that of the case group, which may increase the amount of insulin secreted in their bodies [35]. Additionally, because insulin plays an anabolic role in bone [34], it is possible that this group’s bone mass may be higher than that of the case group. Furthermore, the control group consumed significantly more vitamins and provitamins (A, alpha-carotene, beta-cryptoxanthin, and C), as well as minerals (calcium, phosphorus, magnesium, zinc, manganese, and selenium), than the case group, which consumed more total fat, saturated fat, MUFA, PUFA, and vitamin E. Furthermore, an increase in reactive oxygen species (ROS) levels might produce oxidative stress [36]. Previous research suggests that oxidative stress may contribute to osteoporosis because of its function in chronic inflammation [37]. Antioxidant micronutrients, such as vitamin A, vitamin C, zinc, manganese, and selenium help the body’s defense system against ROS [38] and reduce oxidative stress [36], which contributes to bone health. In addition, the synthesis of collagen and other proteins that comprise the bone structure is influenced by micronutrients, including calcium, magnesium, zinc, and vitamin C [39]. They contribute to the normal development and maintenance of bone mass by performing catalytic functions in the synthesis of bone matrix.

Obesity is exacerbated by an excessive consumption of dietary lipids, such as total fat, polyunsaturated fatty acids, and saturated fatty acids. This, in turn, results in the production of white adipose tissue, which generates pro-inflammatory substances, thereby establishing a protracted inflammatory state [40]. Osteoclasts are known to be primarily activated by pro-inflammatory cytokines like interleukin (IL)-1 and tumor necrosis factor-α (TNF-α), although IL-6 functions in concert with other agents that degrade bone [41]. Therefore, a number of variables that are linked to excess body fat, such as an increase in inflammatory cytokines and a shift in the pattern of adipokine production, negatively impact bone structure [42]. Based on our research, individuals in the lowest third of DII had notably greater consumption of calories, total fat, saturated fat, monounsaturated fat (MUFA), polyunsaturated fat (PUFA), vitamin E, and vitamin D. On the other hand, participants in the highest third of DII had considerably higher intakes of beta-cryptoxanthin, manganese, and selenium. No significant differences were observed among the tertiles for other nutrients. Consequently, the aforementioned mechanisms suggest that a higher ingestion of antioxidants, including beta-cryptoxanthin and manganese, may have a protective effect on bone health in participants who were in the third tertile of DII.

The relationship between DII and the risk of osteoporosis has not been investigated in previous research, to the best of our knowledge. On the other hand, additional research has demonstrated a robust correlation between specific indices and conditions, such as IR [13] and metabolic syndrome [43]. The current investigation also has the advantage of employing a validated food frequency questionnaire to gather data on food consumption. Nevertheless, it is subject to specific limitations. The initial limitation is that insulin scores can only provide an estimate of the total amount of insulinogenic food consumed. They are unable to evaluate the frequency of meals or the composition of food, which could potentially affect insulin response. The second reason is that the case-control design of this study precludes the examination of a coincidental relationship between the risk of osteoporosis and the dietary insulin index. Consequently, it is imperative that additional research corroborates these findings. Two additional drawbacks are recall and choose bias, which are inherent limitations of case-control studies. In order to mitigate recollection bias, this investigation implemented a validated FFQ. Additionally, we examined the FII of dietary items using the FII data from other research [14, 27, 28]. The comparable insulin index of foods must be used for the FII of some foods that were not included in the FFQ because Iranian foods have not yet been measured for FII. This could lead to a slight difference between the FII of foods consumed by individuals and the actual amount of this index in the reference list. Therefore, our findings are impacted by their limits. Another possible drawback of this research might be the absence of information on the length of time spent in the sun, since this could have an impact on bone health. Finally, we only considered the short-term consequence of ingesting foods that increase insulin index, which may increase the need for postprandial insulin and have transitory effects on serum insulin levels [44]. However, an insulinogenic diet may not have a long-term effect on insulin levels. Consequently, it is recommended that additional research be conducted to ascertain the long-term effects of the diet’s propensity to induce insulinemia on serum insulin and bone mass.

## Conclusions

In summary, our results suggested that postmenopausal women may experience an increase in bone mass density by adhering to a diet that is abundant in foods with a higher insulin index. Consequently, it may be essential to consume nutrients that stimulate insulin production in order to prevent osteoporosis. Additional research, particularly prospective cohort studies, is necessary to substantiate these conclusions.

## Data Availability

The datasets used and/or analyzed during the current study are available from the corresponding author on reasonable request.

## Abbreviations

DII: Dietary insulin index
FFQ: Food frequency questionnaire
IR: Insulin resistance
HOMA-IR: Homeostasis model assessment of IR
BMD: Bone mineral density
FII: Food insulin index
GI: Glycemic index
IGF-1: Insulin-like growth factor 1
SHBG: Sex hormone-binding globulin
vBMD: Volumetric bone mineral density
Kg: Kilogram
Cm: Centimeter
BMI: Body mass index
m: Meter
mg: Milligram
g: Gram
µg: Microgram
OR: Odds ratio
SD: Standard deviation
CI: Confidence interval
MET: Metabolic equivalent
kg/m^2^: Kilogram per square meter
OCP: Oral contraceptive pill
Kcal: Kilo calorie
MUFA: Monounsaturated fatty acid
PUFA: Polyunsaturated fatty acid
RAE: Retinol activity equivalent
ROS: Reactive oxygen species
IL: Interleukin
TNF-α: Tumor necrosis factor-α
